# Evaluation of gene expression data generated from expired Affymetrix GeneChip^®^ microarrays using MAQC reference RNA samples

**DOI:** 10.1186/1471-2105-11-S6-S10

**Published:** 2010-10-07

**Authors:** Zhining Wen, Charles Wang, Quan Shi, Ying Huang, Zhenqiang Su, Huixiao Hong, Weida Tong, Leming Shi

**Affiliations:** 1 Division of Systems Biology, National Center for Toxicological Research (NCTR), US Food and Drug Administration (FDA), 3900 NCTR Road, Jefferson, AR 72079, USA; 2 Functional Genomics Core, Beckman Research Institute, City of Hope Comprehensive Cancer Center, 1710 Flower Avenue, Duarte, CA 91010, USA; 3 David Geffen School of Medicine at UCLA, Los Angeles, CA 90095, USA; 4 College of Pharmacy, Western University of Health Sciences, 309 E. Second Street, Pomona, CA 91766, USA; 5 Z-Tech/ICF International at NCTR/FDA, 3900 NCTR Road, Jefferson, AR 72079, USA

## Abstract

**Background:**

The Affymetrix GeneChip^®^ system is a commonly used platform for microarray analysis but the technology is inherently expensive. Unfortunately, changes in experimental planning and execution, such as the unavailability of previously anticipated samples or a shift in research focus, may render significant numbers of pre-purchased GeneChip^®^ microarrays unprocessed before their manufacturer’s expiration dates. Researchers and microarray core facilities wonder whether expired microarrays are still useful for gene expression analysis. In addition, it was not clear whether the two human reference RNA samples established by the MAQC project in 2005 still maintained their transcriptome integrity over a period of four years. Experiments were conducted to answer these questions.

**Results:**

Microarray data were generated in 2009 in three replicates for each of the two MAQC samples with either expired Affymetrix U133A or unexpired U133Plus2 microarrays.  These results were compared with data obtained in 2005 on the U133Plus2 microarray.  The percentage of overlap between the lists of differentially expressed genes (DEGs) from U133Plus2 microarray data generated in 2009 and in 2005 was 97.44%. While there was some degree of fold change compression in the expired U133A microarrays, the percentage of overlap between the lists of DEGs from the expired and unexpired microarrays was as high as 96.99%. Moreover, the microarray data generated using the expired U133A microarrays in 2009 were highly concordant with microarray and TaqMan^®^ data generated by the MAQC project in 2005.

**Conclusions:**

Our results demonstrated that microarray data generated using U133A microarrays, which were more than four years past the manufacturer’s expiration date, were highly specific and consistent with those from unexpired microarrays in identifying DEGs despite some appreciable fold change compression and decrease in sensitivity. Our data also suggested that the MAQC reference RNA samples, stored at -80°C, were stable over a time frame of at least four years.

## Background

As a powerful tool in genomic research, microarray technology has been widely used for simultaneously monitoring expression levels of tens of thousands of genes [1-3]. The Affymetrix GeneChip^®^ system is a commonly used platform for microarray analysis but the technology is inherently expensive. We noticed that unforeseeable changes in experimental planning and execution, *e.g*., unavailability of previously anticipated samples or a shift in research focus could render pre-purchased GeneChip^®^ microarrays unprocessed before their manufacturer’s expiration dates. Therefore, it is important to know whether the expired but expensive GeneChip^®^ microarrays are still useful and reliable in gene expression studies.

A critical goal in the application of microarray technology is to identify differentially expressed genes (DEGs) between sample groups under different conditions. However, several studies showed poor overlaps of DEGs across different platforms or different laboratories using the same sets of RNA samples [4-8], raising concerns on the reliability of microarray technology [9-13] and leading to the launch of the MicroArray Quality Control (MAQC) project [14,15]. The MAQC project systematically evaluated the reliability of microarray technology by profiling two human reference RNA samples (A = Stratagene’s Universal Human Reference RNA; B = Ambion’s Human Brain Reference RNA) in different laboratories using different microarray and QPCR platforms. The MAQC consortium proposed to use fold change ranking combined with a non-stringent *p*-value cutoff for DEG selection and the percentage of overlapping genes (POG) for evaluation of the reproducibility of DEGs between different experiments. Excellent inter-laboratory and inter-platform reproducibility in terms of DEG lists was observed in the MAQC project [15,16].

In the current study, we assessed the consistency between gene expression data generated with expired and unexpired Affymetrix microarrays using the same batches of MAQC samples and a similar data analysis approach as proposed by the MAQC project. Our results showed good reproducibility in terms of DEG lists between the expired and unexpired microarrays. At the same time, we also observed a high level of reproducibility between DEG lists from data generated in 2005 and those newly generated in 2009 with the same type of unexpired U133Plus2 microarrays, suggesting a good degree of stability of the two MAQC reference RNA samples stored at -80°C over a period of four years.

## Results

### Study design and data generation

**Figure 1:**
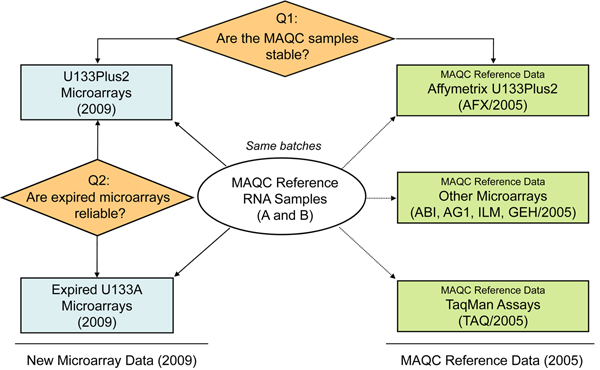
**The workflow of study design and data analysis** New gene expression data were generated in 2009 with expired U133A microarrays and unexpired U133Plus2 microarrays using the same MAQC reference RNA samples and compared to the microarray and TaqMan^®^ gene expression data generated in 2005 by the MAQC project.

We designed and conducted a comparative study to answer the question whether gene expression data generated with Affymetrix U133A microarrays, four years past their expiration date, are useful and reliable. Figure 1 depicts the overview of the study, the experimental procedures, data used, and comparative analyses applied. Two sources of data were used in this study: gene expression data newly generated in 2009 specifically for this study and data generated by the MAQC project in 2005.

The new gene expression data were generated with 12 microarrays (2 types of microarrays × 2 samples × 3 replicates) (Table 1). Three replicates for each of the two MAQC samples (A and B) were profiled in 2009 by using both the expired U133A microarrays (expired in 2004) and the unexpired U133Plus2 microarrays. In addition, gene expression data generated with unexpired U133Plus2 microarrays (AFX), other microarray platforms, and TaqMan^®^ assays by the MAQC project in 2005 were used as references to assess the stability of the MAQC samples stored at -80°C for four years by comparing new microarray data with those obtained four years ago. The MAQC reference data also allowed for further evaluation of the usefulness of the data generated with expired U133A microarrays. 

**Table 1:** New data generated for this study with Affymetrix GeneChip^®^ microarrays

Type of microarrays	Sample^1^	Hybridization name	Scaling factor	Averaged correlation^2^
		A1	4.58	
	A	A2	4.76	0.994
U133A (expired in 2004)		A3	4.29	
	
		B1	4.40	
	B	B2	4.46	0.995
		B3	5.17	

		A1	2.64	
	A	A2	2.95	0.998
U133Plus2		A3	2.72	
	
		B1	2.78	
	B	B2	2.97	0.997
		B3	2.97	

^1^Sample A: Stratagene’s Universal Human Reference RNA; Sample B: Ambion’s Human Brain Reference RNA; ^2^Averaged Pearson correlation coefficient for pair-wise log_2_ intensity comparison across the three replicates.

It should be pointed out that in the current study the observed differences between the expired and unexpired Affymetrix microarrays were confounded with the use of two different types of GeneChip^®^ microarrays, U133A (expired four years ago) and U133Plus2 (unexpired). However, the probe design (probe length and sequence identity) for these two types of GeneChip^®^ microarrays is identical and the consistency of data between the two types of microarrays was demonstrated by the manufacturer (http://media.affymetrix.com/support/technical/technotes/hgu133_p2_technote.pdf). Therefore, the observed difference between expired U133A and unexpired U133Plus2 microarrays can be attributed mainly to the expiration of the former.

### Stability of the two MAQC samples over a period of four years

The two MAQC human reference RNA samples, from the same batches as used in the MAQC project in 2005 but stored at -80°C for over four years, were labeled and hybridized in 2009 on expired U133A and unexpired U133Plus2 microarrays. Before comparing data from the expired and unexpired microarrays, it is necessary to verify the stability of the MAQC samples through assessing the consistency between gene expression data generated in 2005 and in 2009 using the same type of unexpired Affymetrix U133Plus2 microarrays.

**Figure 2:**
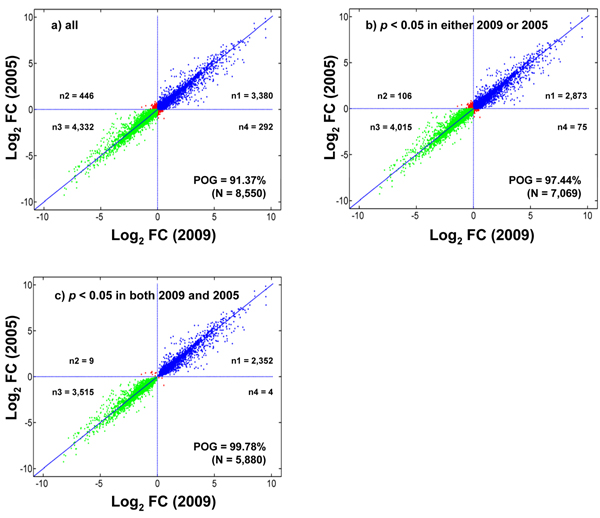
**Comparisons of the log_2_ fold changes detected in 2009 and in 2005 using the same type of U133Plus2 microarrays** (a) all 8,550 common genes; (b) the 7,069 genes with *p* < 0.05 in either 2009 or 2005; and (c) the 5,880 genes with *p* < 0.05 in both 2009 and 2005. Fold changes were generated by comparing sample B to sample A, *i.e*., B/A. The blue and green dots indicated the up- and down-regulated genes, respectively. The red dots were the genes with reverse regulation directionalities in 2009 and in 2005. Under the three scenarios (a, b, and c), the overlap of differentially expressed genes between 2009 and 2005 is 91.37%, 97.44%, and 99.78%, respectively, suggesting a high degree of stability of the two MAQC reference RNA samples.

Figure 2 shows the correlation of log_2_ fold changes observed in 2009 and in 2005 with unexpired U133Plus2 microarrays. The correlations are shown in Figures 2a, 2b, and 2c for the 8,550 genes commonly probed by multiple microarray platforms, the 7,069 genes with a *p* < 0.05 in either of the two data sets (2009 and 2005), and the 5,880 genes with a *p* < 0.05 in both data sets, respectively. It can be seen from Figure 2 that most genes showed similar log_2_ fold changes. Under the three gene selection scenarios (a, b, and c) with increasing stringency, the overlap of DEGs between 2009 and 2005 was 91.37%, 97.44%, and 99.78%, respectively, suggesting a reasonably high degree of stability of the two MAQC samples over a period of four years. Thirteen genes showed opposite regulation directionalities between the data sets generated in 2005 and in 2009 (Figure 2c). It should be pointed out that DEG concordance is only a surrogate of sample stability and more direct measurement of sample stability is warranted in future studies.

### Repeatability of intensity data among sample replicates on expired microarrays

**Figure 3:**
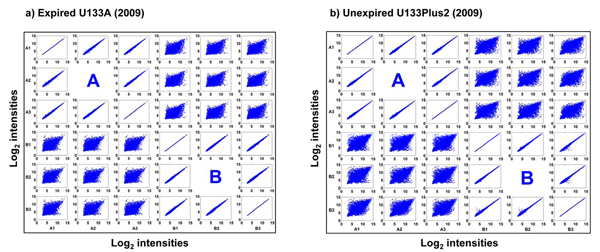
**Correlations of log_2_ intensities generated in 2009 among the replicates of samples A and B** (a) expired U133A microarrays and (b) unexpired U133Plus2 microarrays. Each scatterplot represents the comparison of log_2_ intensities of 8,550 common genes from two hybridizations.

The probe-level raw intensity data were first summarized and normalized with the RMA algorithm [17] and then transformed to log_2_ scale. Figures 3a and 3b show the correlation of the log_2_ gene expression intensities for the 8,550 common genes between replicates on the expired U133A and unexpired U133Plus2 microarrays, respectively. For the expired U133A microarrays (Figure 3a), the replicates of the same sample showed a high level of correlation similar to what was observed for the unexpired U133Plus2 microarrays (Figure 3b). More quantitatively, the average correlation coefficients of the log_2_ intensities among the replicates of sample A were 0.994 and 0.998 for the expired and unexpired microarrays, respectively. For sample B, the corresponding average correlation coefficients were 0.995 and 0.997 from the expired and unexpired microarrays, respectively (Table 1). These results demonstrated a high level of intra-sample repeatability of absolute gene expression data from expired U133A microarrays.

### Comparison of log_2_ fold changes detected with expired U133A and unexpired U133Plus2 microarrays

**Figure 4:**
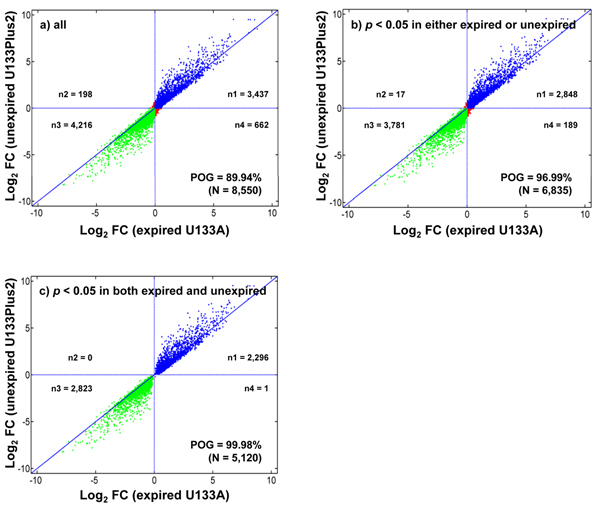
**Comparisons of log_2_ fold changes detected with expired U133A and unexpired U133Plus2 microarrays** (a) all 8,550 common genes; (b) the 6,835 genes with *p* < 0.05 in either expired U133A microarrays (2009) or unexpired U133Plus2 microarrays (2009); and (c) the 5,120 genes with *p* < 0.05 in both expired U133A (2009) and unexpired U133Plus2 (2009) microarrays. Fold changes were generated by comparing sample B to sample A, *i.e*., B/A. The blue and green dots indicated the up- and down-regulated genes, respectively. The red dots were the genes with reverse regulation directionalities. Under the three scenarios (a, b, and c), the overlap of differentially expressed genes between expired U133A (2009) and unexpired U133Plus2 (2009) microarrays is 89.94%, 96.99%, and 99.98%, respectively, indicating a high degree of consistency between differential gene expression data generated from expired U133A and unexpired U133Plus2 microarrays. Note that the fold changes measured with expired U133A microarrays exhibited some degree of compression (*i.e*., with smaller absolute values) when compared to those obtained with unexpired U133Plus2 microarrays.

To address the issue of using expired microarrays, the log_2_ fold changes of data obtained with the expired U133A microarrays and the unexpired U133Plus2 microarrays were compared (Figure 4). The log_2_ fold changes for most genes were consistent in the direction of regulation (down or up). However, there was a slight fold change compression [18] for the data obtained with the expired U133A microarrays. That is, the magnitude of differential expression (fold change) for the same gene measured from the expired U133A microarrays (the X axis) was lower than that from the unexpired U133Plus2 microarrays (the Y axis). 

The percentage of overlapping genes with the same directional change in expression was 89.94% (7,690 out of 8,550 common genes) when no *p*-value cutoff was applied to either experiment (Figure 4a). The overlap increased to 96.99% (6,629 out of 6,835 genes) when genes were retained for comparison as long as a *p* < 0.05 criterion was met in either one of the two experiments (Figure 4b), a fair scenario for comparing two data sets. When the comparison was restricted to the subset of 5,120 genes that were simultaneously detected to be significantly differentially expressed (*p* < 0.05) in both experiments, the overlap increased to 99.98%. That is, only one gene showed opposite regulation directionalities between expired U133A microarrays and unexpired U133Plus2 microarrays. These results suggested that the DEGs detected by the expired U133A microarrays were highly consistent with those by the unexpired U133Plus2 microarrays. 

### Comparison between expired U133A microarrays with multiple microarray platforms used in the MAQC project

**Figure 5:**
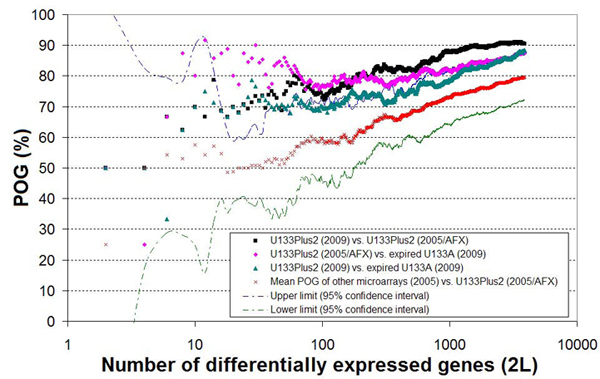
**Concordances of different microarrays in terms of percentage of overlapping genes (POG)** The x-axis represents the number of genes (2*L*) selected as differentially expressed, and the y-axis represents the overlap (%) of two gene lists selected from the two experiments under comparison. All of the genes in the two compared experiments were first filtered with a *p* < 0.05 cutoff and then ranked by fold changes in up- and down directions. Next, the top *L* genes with the largest fold changes were selected as differentially expressed from each direction and the DEG list containing 2*L* genes from each experiment was used for cross-experiment comparisons. *L* was increased from one to the lower number of genes in both regulation directions with a step of one. The red crosses represent the reference line, *i.e.*, the mean POG of DEG lists in four comparisons of microarray reference data groups in MAQC project: ABI, AG1, ILM, and GEH versus AFX. The dashed and dotted green and blue markers showed the lower limit and the upper limit of the 95% confidence intervals, respectively, for the reference line.

Microarray data generated in 2005 with the same MAQC samples from five platforms, Applied Biosystems (ABI), Affymetrix U133Plus2 (AFX), Agilent (AG1), Illumina (ILM), and GE Healthcare (GEH) by the MAQC project [15] were used as references to further assess the usefulness of the expired U133A microarrays. To assess the concordance between two experiments, we first used a *p* < 0.05 cutoff to eliminate genes separately in each experiment and then rank-ordered the remaining genes by log_2_ FCs. In one experiment, genes ranked higher in either the up- or down-regulation direction are selected as DEGs first. Figure 5 shows the relationship between the POG and the number of selected DEGs, wherein genes with smaller FCs are selected when more genes are chosen as DEGs. The red line represented the average POG between DEG lists of each of the four reference microarray data sets in the MAQC project (ABI, AG1, ILM, and GEH) and the reference Affymetrix U133Plus2 (AFX) data, all generated in 2005. This reference line and its confidence limits can be used to judge the statistical significance between any two experiments, *e.g*., between expired U133A microarrays and unexpired U133Plus2 microarrays. 

The concordance between DEG lists selected from expired U133A microarrays and unexpired U133Plus2 microarrays (2009 or 2005) were about 70% when the number of selected DEGs was as few as 10. These POG numbers increased to near 90% when more genes were selected as differentially expressed. For the same number of DEGs, the POGs between expired U133A (2009) and unexpired U133Plus2 (2009) microarrays, and between expired U133A (2009) and the reference U133Plus2 (AFX in 2005) microarrays were on the upper confidence limit of the reference line. This means that compared to data from the reference AFX microarrays (2005), the DEGs generated with expired U133A microarrays (2009) are more consistent than those from other unexpired, but different microarray platforms (ABI, AG1, ILM, and GEH) in 2005. In addition, the POG between the unexpired U133Plus2 (2009) and the reference AFX microarrays (2005) was the highest, again, suggesting that the MAQC samples were relatively stable. 

### Comparison between expired U133A microarrays and TaqMan^®^ assays

**Figure 6:**
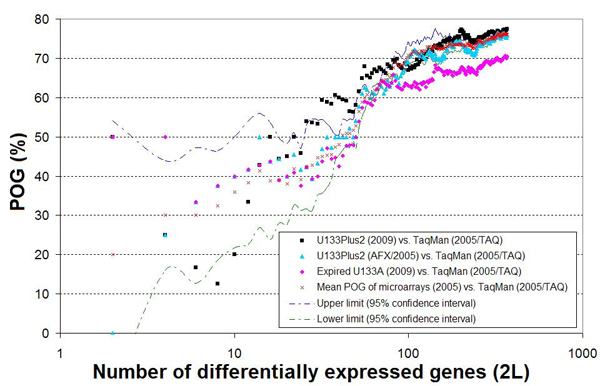
**Concordance between TaqMan^®^ assays and microarray data in terms of percentage of overlapping genes (POG)** The x-axis represents the number of genes (2*L*) selected as differentially expressed, and the y-axis represents the overlap (%) of two gene lists selected from the two experiments under comparison. See notes under Figure 5 for more information.

TaqMan^®^ assays have been widely used in gene expression profiling and are generally considered as a good reference for evaluating other gene expression techniques. In this study, the TaqMan^®^ gene expression data generated by the MAQC project in 2005 using the same MAQC samples [15,19] were used to further assess the usefulness of the expired U133A microarrays based on data from the 813 genes that were probed by both the microarrays and TaqMan^®^ assays. The DEG lists obtained from expired U133A microarrays (2009), unexpired U133Plus2 microarrays (2009), and microarrays from ABI, AFX, AG1, ILM, and GEH (2005) were separately compared with those from the TaqMan^®^ assays performed in 2005 [19]. The mean POG of the DEG lists between the TaqMan^®^ assays and the microarray platforms (ABI, AFX, AG1, ILM, and GEH) was shown as a reference line (the red crosses in Figure 6). The POG between the expired U133A microarrays (2009) and the TaqMan^®^ assays (pink diamonds) was close to the reference line when the number of DEGs was as low as 40 but was about 10% lower than the reference line when all genes meeting the *p* < 0.05 criterion were selected (a drop from ~77% to ~70%). In addition, the POG between unexpired U133Plus2 microarrays (2009) and TaqMan^®^ assays (black squares) and the POG between the AFX (2005) and TaqMan^®^ assays (sky blue triangles) were higher than, or at the same level as, the reference line. That is, compared to unexpired microarrays, expired U133A microarrays exhibited a slightly decreased ability to detect differential gene expression, mainly due to the fold change compression that made the rankings of genes with smaller fold changes more variable. 

### Characteristics of gene expression data measured on unexpired U133Plus2 and expired U133A microarrays

The lower sensitivity of the expired U133A microarrays in detecting differential gene expression could be explained by a decrease in microarray signal or an increase in the scaling factor (Table 1). The average scaling factor was 4.61 for the expired U133A microarrays and 2.83 for the unexpired U133Plus2 microarrays. The higher scaling factor for the expired microarrays indicates that compared to the unexpired microarrays, the quality of hybridization signals from the expired microarrays was somewhat compromised, leading to a decrease in sensitivity. 

**Figure 7:**
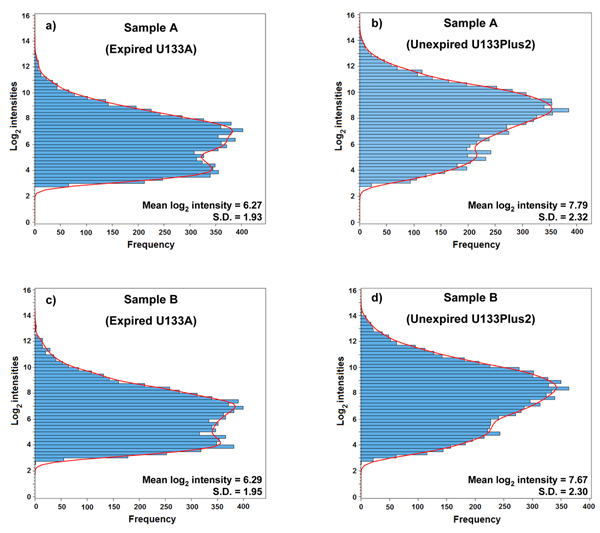
**Distribution of the log_2_ intensities of 8,550 common genes** Sample A hybridized on expired U133A (a) and unexpired U133Plus2 (b) microarrays; Sample B hybridized on expired U133A (c) and unexpired U133Plus2 (d) microarrays. For each gene, the log_2_ intensities of the three technical replicates were averaged. Based on microarray data generated in 2009.

**Figure 8:**
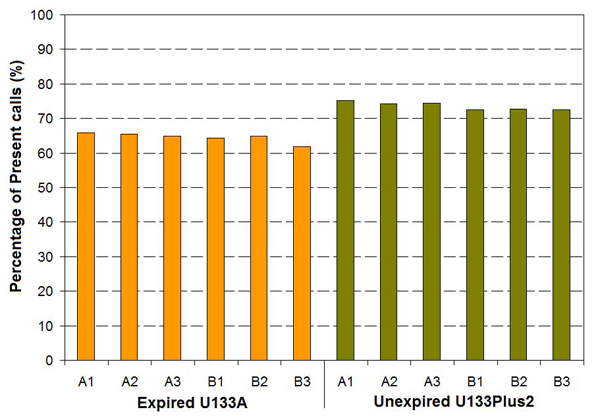
**Percentage of Present calls based on 8,550 common genes** There is a significant difference in the percentage of Present calls between unexpired U133Plus2 microarrays (73.6%) and expired U133A microarrays (64.5%). Based on microarray data generated in 2009.

Figure 7 shows the distributions of log_2_ intensities for 8,550 common genes when the MAQC samples were analyzed with the expired U133A and the unexpired U133Plus2 microarrays. Compared to the U133Plus2 microarrays, which showed a mean log_2_ intensity of 7.79 for sample A (Figure 7b) and 7.67 for sample B (Figure 7d), the expired U133A microarrays exhibited a significant decrease in mean log_2_ intensity, *i.e*., 6.27 for sample A (Figure 7a) and 6.29 for sample B (Figure 7c). The standard deviations of log_2_ intensity for unexpired U133Plus2 microarrays (2.32 for sample A and 2.30 for sample B) are also higher than those for expired U133A microarrays (1.93 for sample A and 1.95 for sample B), indicating a decrease in the ability of expired microarrays to distinguish differences of expression levels among genes in the same sample. In addition, as shown in Figure 8, there is a significant difference in the percentage of Present calls between unexpired U133Plus2 (73.6%) and expired U133A (64.5%) microarrays, also suggesting a significant loss of detection signals for the expired U133A microarrays.

## Discussion

Many users of microarrays face time pressures to complete their studies while staying within the expiration dates on the microarrays. Moreover, it is important to understand the stability of RNA samples after years of storage. Our current study investigated the stability of two MAQC human reference RNA samples and examined the reliability of expired Affymetrix U133A microarrays in gene expression analyses. We compared the gene expression data of expired and unexpired microarrays from three aspects: absolute expression intensities, log_2_ fold changes, and the POG of DEG lists, using data newly generated in 2009 with the same pair of reference RNA samples used in 2005 by the MAQC project. In addition, the gene expression data generated using different platforms (ABI, AFX, AG1, ILM, GEH, and TaqMan^®^) in 2005 by the MAQC project were used as references to evaluate the reliability of the expired U133A microarrays and also to assess the stability of the MAQC samples.

Based on the log_2_ fold changes, microarray data generated in 2009 were highly consistent (97.44%) with those generated by the MAQC project in 2005 using exactly the same type of unexpired Affymetrix U133Plus2 microarrays and with the same pair of RNA samples that had been stored at -80°C for over four years (Figures 2 and 5). Moreover, when compared with the TaqMan^®^ assay data generated by the MAQC project in 2005, the two sets of data (2009 and 2005) from unexpired U133Plus2 microarrays showed similar results (Figure 6). Our comparative analyses indicated that the microarray data generated in 2009 from the same pair of MAQC RNA samples were reliable and that the RNA samples used in this study were reasonably stable after a period of at least four years. Therefore, it is a reasonable strategy to use these samples for assessing the reliability of the expired U133A microarrays.

Despite appreciable fold change compression, 96.99% genes identified from expired U133A microarrays as differentially expressed were consistent in the direction of regulation with those from unexpired U133Plus2 microarrays when the comparison was restricted to those genes with *p* < 0.05 in at least one experiment (Figure 4b). These results suggested that the DEGs detected by the expired U133A microarrays were highly compatible with those identified by the unexpired U133Plus2 microarrays. Our comparative analyses also showed that the DEG lists generated with the expired U133A microarrays were more reproducible than those from other unexpired microarray platforms (ABI, AG1, ILM, and GEH) in 2005 when compared to the DEG lists from unexpired U133Plus2 microarrays in 2005 (AFX) and in 2009 (Figure 5). That is, the level of concordance between expired and unexpired Affymetrix microarrays is higher than the level of concordance between unexpired Affymetrix microarrays and microarrays from other different platforms.

The POG of the DEG lists from the expired U133A microarrays and the TaqMan^®^ assays was ~10% lower than the average POG of the DEG lists between the unexpired microarrays and TaqMan^®^ assays (Figure 6). The number of DEGs detected by the expired U133A microarrays was lower than that by the unexpired U133Plus2 microarrays and the magnitude of the differential gene expression (fold change) detected by expired U133A microarrays was also lower. 

## Conclusions

In this study, two human reference RNA samples, established and used in the MAQC project four years ago, were used to assess a) the stability of these reagents and b) the reliability of data generated on Affymetrix’s U133A microarrays expired four years ago. By comparing the detected log_2_ fold changes and the POG between the DEG lists from unexpired Affymetrix U133Plus2 microarrays in 2005 and in 2009, we first demonstrated that the reference RNA samples were relatively stable and in reasonably good quality. Comparative analyses showed a reasonable level of consistency in DEG lists between the expired and unexpired microarrays. Comparison between DEG lists from expired U133A microarrays and TaqMan^®^ assays showed a decrease in sensitivity of expired U133A microarrays by approximately 10%. In addition to possible sample-related reasons, the increase of scaling factors, the shrinkage of the distribution of detected raw intensities, and the lower percentage of Present calls indicate a decrease in detection sensitivity for the expired microarrays. Our results suggested that the Affymetrix U133A microarrays expired four years ago generated useful, but somewhat less sensitive, data for identifying genes differentially expressed. It is warranted to continue to monitor the stability and integrity of the MAQC reference RNA samples because of their wide applications by the gene expression community.

## Methods

### Microarray data generated in 2009 on U133Plus2 and expired U133A microarrays

The two reference RNA samples A (Stratagene’s Universal Human Reference RNA) and B (Ambion’s Human Brain Reference RNA), which were established and extensively profiled in the MAQC project in 2005 [15], were purchased in 2009 from Agilent and Life Technologies, respectively, to generate new microarray gene expression data specifically for this study. The samples had been stored under -80°C at their respective vendor’ freezer since 2005, and were shipped with dry ice from the vendors to the microarray laboratory of Dr. Charles Wang at UCLA. The same batches of the two samples as those used in the MAQC project in 2005 were used in this study. Upon receiving RNA samples in 2009, the microarray laboratory evaluated and confirmed their quality using the RNA 6000 LabChip and Agilent 2100 Bioanalyzer. The RNA Integrity Number (RIN) for each sample was around 8.0. Biotinylated cRNA samples were prepared according to the standard Affymetrix GeneChip^®^ protocol. One µg of total RNA from each sample, along with poly A spikes (labeling control), were converted to double-stranded cDNA with GeneChip^®^ One-Cycle cDNA Synthesis Kit (Affymetrix, Santa Clara, CA). After second-strand synthesis, the cDNA was purified with the GeneChip^®^ Sample Cleanup Module (Affymetrix). The resulting double-stranded DNA was then used to generate multiple copies of biotinylated cRNA by *in vitro* transcription with the GeneChip^®^ 3’-Amplification Reagent Kit for IVT Labeling (Affymetrix). The A260/A280 ratio and yield of each of the cRNAs were recorded. For each sample, 10 µg of biotinylated cRNA spiked with bioB, bioC, bioD and cre (Hybridization Control) was hybridized to a GeneChip^®^ microarray for 16 hours at 45°C. Following hybridization, all microarrays were washed and stained in an Affymetrix GeneChip^®^ Fluidics Station. Stained microarrays were scanned with an Affymetrix GeneChip^®^ Scanner 3000. Quality checks and data analyses were carried out using Affymetrix GeneChip^®^ Operating Software (GCOS) and Quality Reporter. Each of the two samples was processed in triplicate, starting from independent labeling reactions with a new aliquot of the reference sample from the same tube, a) on Affymetrix Human Genome U133A microarrays that were four years ago past the manufacturer’s expiration dates and b) on unexpired Human Genome U133Plus2 microarrays. The expired U133A microarrays were stored in a refrigerator at 4°C, as recommended by the manufacturer. In total, gene expression data were collected from 12 (2 types of microarrays × 2 samples × 3 replicates) microarrays. The comparisons among the microarrays were based on 8,550 common genes, which represented the intersection of the 12,091 genes commonly probed by the microarray platforms used in the MAQC project [15] and the genes probed by the U133A microarray platform used in this study. It should be pointed out that from 2005 to 2009 Affymetrix implemented some changes such as the sources of labeling kits into its GeneChip^®^ product pipeline. Consequently, comparing gene expression data from the same sample in 2005 and 2009 at the intensity level is not meaningful, because it has been demonstrated that intensity data are highly dependent on the labeling protocols among many other factors.

### Microarray and TaqMan^®^ data generated by the MAQC project in 2005

The gene expression data generated from the two samples by the MAQC project with five commercial microarray platforms, Applied Biosystems (ABI), Affymetrix (AFX), Agilent (AG1), Illumina (ILM), and GE Healthcare (GEH), and with the TaqMan^®^ (TAQ) assays were obtained from the MAQC project (GSE5350) and used in this study as references for comparison. For fair comparisons, the first three replicates reported by the MAQC project from each platform’s first test site were used in this study. For cross-platform comparison, the MAQC consortium mapped all the probe sequences of the five high-density microarray platforms to the RefSeq human mRNA database [20] and selected 12,091 common Entrez genes that were uniquely matched by 12,091 probes/probesets on each of the five microarray platforms, resulting in a “one-probe-to-one-gene” cross-platform mapping table with 12,091 genes and the probe/probeset identifiers from multiple microarray platforms and TaqMan^®^ assays (http://www.nature.com/nbt/journal/v24/n9/extref/nbt1239-S5.txt) [15]. The probeset IDs from the U133A platform used in this study were mapped to this “one-probe-to-one-gene” cross-platform mapping table, resulting in 8,550 “common” genes that are probed by all six microarray platforms (including the five microarray platforms used in MAQC project in 2005 and the U133A platform used in the current study). Among the 8,550 common genes, 813 were also assayed with TaqMan^®^ and were used for comparing microarrays with TaqMan^®^ assays.

### Computational tools for data analysis

The microarray data generated in 2009 and the data from the MAQC project generated in 2005 were imported to ArrayTrack™ 3.5.0 [21], a software system developed by the US Food and Drug Administration’s National Center for Toxicological Research for microarray data management, analysis, visualization, and interpretation (http://www.fda.gov/ScienceResearch/BioinformaticsTools/Arraytrack/). Unless stated otherwise, the probe level data were summarized into probeset level data using RMA [17] before statistical testing; these calculations were performed within ArrayTrack that has an interface with Bioconductor 2.4. Note that RMA summarization was performed separately on the 6 unexpired U133Plus2 microarrays and on the 6 expired U133A microarrays hybridized in 2009. Data collected by the MAQC project in 2005 were treated similarly, with RMA summarization performed on the 6 microarrays (3 replicates per sample) from each platform.

The scaling factor of each microarray was calculated with the Affy package in Bioconductor 2.4 within R 2.9. The comparisons of log_2_ intensities, log_2_ fold changes, and the POGs were conducted within MATLAB (MathWorks, Natick, MA) and the corresponding homemade source codes for these analyses are available upon request.

When probe-level raw expression data are summarized into probeset expression values with MAS5, a trimmed mean of the normalized data is scaled to a default value *(e.g*., 500 in the Affy package in Bioconductor 2.4) by using a scaling factor [22]. The value of the scaling factor is often used as a metric to assess the quality of hybridization and the consistency across multiple microarrays. A scaling factor closer to 1 represents better hybridization signal quality.

### Statistical methods for identifying DEGs

Welch’s *t*-test is one of the common statistical methods for gene selection. The *p*-value calculated by Welch’s *t*-test is usually used to measure the statistical significance of a gene differentially expressed in the two groups of samples being compared. In our study, the *p-*value is directly used for gene filtering without multiple-testing correction. A gene was considered as significantly differentially expressed if its *p*-value was less than 0.05. 

The fold change (FC) of gene expression intensities represents to what extent a gene is differentially expressed between two groups of samples. After filtering genes with a *p* ≥ 0.05 criterion, the remaining genes were ranked by their fold changes (reference sample B/reference sample A). Then, at each given cutoff a list of the number of genes considered to be differentially expressed genes was generated for subsequent comparisons. 

### Percentage of overlapping genes (POG)

The genes contained in the two compared DEG lists with the same direction of regulation (i.e., decreased or increased) were considered to be overlapped genes. The percentage of the overlapping genes (POG) [14,15,23] among the total number of genes in one DEG list was calculated and used to measure the consistency between the two DEG lists by using the following equation:

,

where *DD* and *UU* are, respectively, the number of commonly down- and up-regulated genes. *L* is the number of genes with down- or up-regulation. Similar to previous studies [14,15,23] we selected an equal number of down- and up-regulated genes in each of the two DEGs lists. To obtain an overall picture on the overlap between two experiments, we evaluated the POG at a wide range of gene selection criteria by changing *L* from one (corresponding to the largest FC) to the lower number of genes in the two regulation directions (corresponding to the smallest FC) with a step of one.  See Figures 5 and 6 for more information. 

All microarray data specifically generated for this study are available through the National Center for Biotechnology Information’s Gene Expression Omnibus (series accession number: GSE23906). 

## Competing interests

The authors declare that they have no competing interests.

## Authors’ contributions

LS conceived the study design. CW, QS, and YH generated the microarray data. ZW, QS, YH, ZS and LS carried out data analysis. ZW, CW, QS, HH, WT and LS prepared the manuscript. All authors read and approved the final manuscript.
